# Early Warning Indicators for HIV Drug Resistance in Cameroon during the Year 2010

**DOI:** 10.1371/journal.pone.0036777

**Published:** 2012-05-17

**Authors:** Serge C. Billong, Joseph Fokam, Armand S. Nkwescheu, Etienne Kembou, Pascal Milenge, Zephirin Tsomo, Grace Ngute Dion, Avelin F. Aghokeng, Eitel N. Mpoudi, Peter M. Ndumbe, Vittorio Colizzi, Jean B. Elat Nfetam

**Affiliations:** 1 National HIV Drug Resistance Surveillance and Prevention Working Group (HIVDR-WG), National AIDS Control Committee (NACC), Yaounde, Cameroon; 2 Central Technical Group (CTG), National AIDS Control Committee (NACC), Ministry of Public Health, Yaounde, Cameroon; 3 Faculty of Medicine and Biomedical Sciences (FMBS) of the University of Yaoundé 1, Yaounde, Cameroon; 4 Chantal BIYA International Reference Centre (CIRCB) for Research on HIV/AIDS Prevention and Management, Yaounde, Cameroon; 5 Division of Operational Health Research (DROS), Ministry of Public Health, Yaounde, Cameroon; 6 World Health Organisation (WHO), Regional Office for Africa (AFRO), National Office, Yaoundé, Cameroon; 7 Approved Treatment Centre (ATC), Yaounde Central Hospital, Yaounde, Cameroon; 8 Approved Treatment Centre (ATC), Yaounde General Hospital, Yaounde, Cameroon; 9 Laboratory of Virology, Centre de Recherche en Maladies Emergentes et Ré-émergentes (CREMER), Yaounde, Cameroon; 10 Faculty of Health Sciences (FHS), University of Buea, Buea, Cameroon; 11 University of Rome “Tor Vergata”, Faculty of Sciences, Department of Biology, Rome, Italy; 12 University of Rome “Tor Vergata”, UNESCO Chair of Biotechnology, Rome, Italy; National Microbiology Laboratory, Canada

## Abstract

**Background:**

Rapid scale-up of antiretroviral therapy (ART) in resource-limited settings is accompanied with an increasing risk of HIV drug resistance (HIVDR), which in turn could compromise the performance of national ART rollout programme. In order to sustain the effectiveness of ART in a resource-limited country like Cameroon, HIVDR early warning indicators (EWI) may provide relevant corrective measures to support the control and therapeutic management of AIDS.

**Methods:**

A retrospective study was conducted in 2010 among 40 ART sites (12 Approved Treatment Centers and 28 Management Units) distributed over the 10 regions of Cameroon. Five standardized EWIs were selected for the evaluation using data from January through December, among which: (1) Good ARV prescribing practices: target = 100%; (2) Patient lost to follow-up: target ≤20%; (3) Patient retention on first line ART: target ≥70%; (4) On-time drug pick-up: target ≥90%; (5) ARV drug supply continuity: target = 100%. Analysis was performed using a Data Quality Assessment tool, following WHO protocol.

**Results:**

The number of sites attaining the required performance are: 90% (36/40) for EWI_1_, 20% (8/40) for EWI_2_; 20% (8/40) for EWI_3_; 0% (0/37) for EWI_4_; and 45% (17/38) for EWI 5. ARV prescribing practices were in conformity with the national guidelines in almost all the sites, whereas patient adherence to ART (EWI_2_, EWI_3_, and EWI_4_) was very low. A high rate of patients was lost-to-follow-up and others failing first line ART before 12 months of initiation. Discontinuity in drug supply observed in about half of the sites may negatively impact ARV prescription and patient adherence. These poor ART performances may also be due to low number of trained staff and community disengagement.

**Conclusions:**

The poor performance of the national ART programme, due to patient non-adherence and drug stock outs, requires corrective measures to limit risks of HIVDR emergence in Cameroon.

## Introduction

With a general population estimated at 19,401,000 inhabitants in 2010, Cameroon had close to 560,300 adults and children living with HIV/AIDS, among which 249,341 were eligible to receive highly active antiretroviral therapy (HAART) based on the World Health Organisation (WHO) criteria for eligibility to treatment (CD4<350 cells/µL) [1]–[3]. Already 35.6% (89,455) of patients eligible for treatment were receiving antiretroviral therapy (ART) in the 145 treatment centers [HIV Approved Treatment Centers (ATC), and HIV Management Units (HMU)] nationwide [4]–[5]. Despite the effectiveness of HAART in reducing morbidity and mortality associated with AIDS, the ongoing scale-up of antiretrovirals (ARV) is inevitably associated with a high risk of HIV drug resistance (HIVDR) to ARVs commonly used in the country [6]–[7]. Since such practices might compromise the performance of the national ART programme, HIVDR surveillance and prevention are of paramount importance [8]–[11].

Surveillance and prevention of HIVDR was launched in Cameroon with three main components: (1) Evaluation of early warning indicators (EWIs) for HIVDR; (2) Evaluation of the threshold of transmitted HIVDR; and (3) Monitoring of HIVDR among patients on ART [5]. These efforts resulted into recent studies conducted by *Aghokeng et al.* and *Kouanfack et al.*, showing evidence of low to moderate levels of transmitted and acquired drug resistance in adults living with HIV/AIDS in Cameroon [12], [13]. More recently, *Fokam et al.* showed a low (4.9%) and very high (90%) levels of pediatric HIVDR, respectively among children naïve to ART and those failing first line treatment, with a median time-to-treatment failure of <2 years [14]. Despite the increasing awareness in Cameroon about HIVDR, and the development of cost-effective HIVDR genotyping assays by *Yang et al.* and by *Fokam et al.*, respectively in 2010 and 2011 [15]–[16], these reference assays are still unaffordable to a great majority of patients. Due to the known limited affordability to such reference laboratory equipment (HIV viral load and HIVDR testing) [4], WHO strongly recommends the prevention of HIVDR using EWIs as the less costly and rapid intervening population-based approach to support and sustain the efficacy of current treatment guidelines [8], [17]. To this effect, WHO proposes six strongly recommended and two optional EWIs, among which countries are advised to select at least 4 for their national HIVDR prevention activities [17]. Based on the contextual feasibility, five strongly recommended EWIs were chosen by Cameroon [5]:

- EWI_1_: *“Good ARV prescribing practices”* (Percentage of patients initiated on an appropriate first line ARV drug regimen). Numerator: Number of individuals initiating ART at the site who are prescribed a standard or otherwise appropriate first-line regimen during the selected time period. Denominator: Number of individuals starting ART during the selected time period. The acceptable target performance: 100%.
- EWI_2_: “*Patient lost to follow-up”* (Percentage of patient lost to follow-up after 12 months of enrolment to ART). Numerator: Number of individuals initiating ART in a selected time period who were not seen at the clinic or pharmacy ≥90 days after the date of their last missed appointment or drug pick-up that occurred within their first 12-months of ART, and who are not known to have transferred out or died. Denominator: Number of individuals starting ART during a selected time period. The acceptable target performance ≤20%.
- EWI3: *“Patient retention on appropriate first line ART”* (Percentage of patient retention on appropriate first line ART after 12 month of treatment). Numerator: Number of individuals initiating first-line ART during a selected period of time who are, 12 months from ART start, still on first-line ART (this includes substitutions of one appropriate first-line regimen for another, but not substitutions of dual- or monotherapy or a inappropriate three-drug regimen). Denominator: Number of individuals starting ART during a selected time period or, in sites where data are available, that number minus the number of individuals starting ART in that time period who transferred out during the 12 months after starting ART. Individuals who died, stopped ART, switched to second-line ART, or were lost to follow-up must be included in the denominator. The acceptable target performance ≥70%.
- EWI_4_: *“On-time ARV drug pick-up”* (Percentage of on-time ARV drug pick-up by the patient). Numerator: number of individuals who have picked up all their prescribed ARV drugs on time during the selected time period. Denominator: number of individuals classified as “on ARV drugs” during the selected time period. The acceptable target performance ≥90%.
- EWI_5_: *“Drug supply continuity”* (Percentage of ARV drug supply continuity at the site pharmacy). Numerator: Number of months or quarters in the year in which there were no ARV drug stock outages for any ARVs in any of the standard ART regimens supplied by the site or the pharmacy at which the site’s patients pick up ARV drugs. Denominator: 12 months (or 4 quarters). The acceptable target performance: 100%.

The objectives of our study is to evaluate the levels of these five chosen HIVDR EWIs in ART sites all over Cameroon, in order to identify potential strengths and weaknesses of the national ART programme, and to inform the relevant evidence-based policies necessary to improve the quality of clinical healthcare, the drug supply/management system, and patient adherence; with the ultimate goal of minimizing the development and the spread of preventable HIVDR in the country.

## Methods

### Study Design

A descriptive, longitudinal and retrospective survey was conducted in 2010 among 40 ART sites, 12 Approved Treatment Centers (ATC) and 28 HIV Management Units (HMU), distributed over the Cameroon national territory. The sample size for EWI_1_ to EWI_4_ at each ART site was calculated based on the WHO sampling method [5], [17], a formula used to compare an observed percentage to a theoretical percentage (threshold of EWIs): N  =  (Z^2^ × P × Q)/d^2^; with N being the minimum sample size, Z at an confidence interval of 95% (Z = 1.96), P as the expected percentage of retention on first line ART (≥70%), Q as 1- P (30%), and D as the degree of accuracy ( = 7%). EWI_5_ was calculated using the number of months in the year during which there were no ARV drug stock outages for any ARVs in any of the standard ART regimens.

Ethical clearance for the study was obtained from the Cameroon National Ethics Committee (Authorization N°034/NEC/SE). Confidentiality was respected during abstraction of data by the use of specific identification code for each enrolled patient number. Since our study was conducted retrospectively, data were collected from medical/ART and pharmacy registers available at the ART sites. Thus, informed consent from the participants was not required.

As per WHO-recommendations, heads of ART sites involved in the survey and their respective data managers were preliminarily trained on the relevance of HIVDR EWIs and on the methodology for data collection and results interpretation. At the level of each ART site, the five EWIs were collected using WHO-standardized data collection forms. In detail, the first three EWIs were obtained by abstraction of cumulative cohort data from patient medical records/ART registers, while the two other EWIs were abstracted from the pharmacy registers. A description of each ART site was done, taking into consideration the level of the HIV/AIDS clinic (ATC or HMU), the number of staff trained and involved in the routine management of people living with HIV. Data were collected among patients newly enrolled on ART (starting from the month of January 2009, until completing the required sample size) and followed-up throughout the first year of ART, with an additional three months extension, necessary when monitoring patients lost to follow-up at the ART site.

### Data Validation, Analysis and Interpretation

Data validation was performed onsite using hard copies of collected data and the ART registers; these data were then centralized at the national level and entered into a standardized electronic excel sheet. A second validation using the electronic and the hard data sheet was done at the central level to ensure reliability and coherence. Validated data were then analyzed per each site and then globally (nationwide), using a WHO routine data quality assessment tool (RDQA) designed specifically for such surveys. Results were automatically generated in the Excel spread sheet.

An EWI attaining the acceptable or required target was classified as ***“Good Performance”***, and any EWI unable to reach the acceptable or required target was classified as ***“Poor Performance”***.

## Results

### Description of the Surveyed Antiretroviral Therapy (ART) Sites

**Figure 1:**
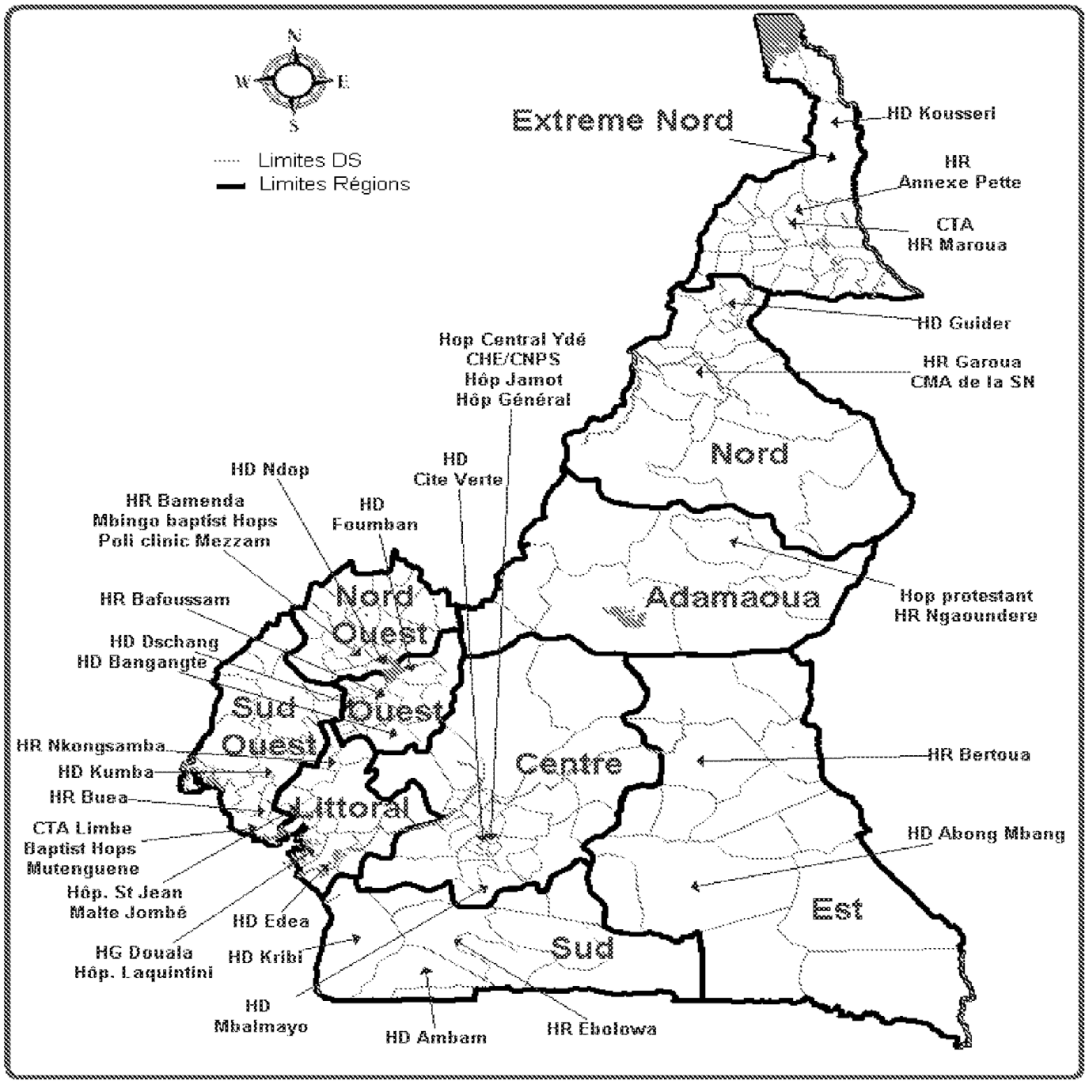
Geographical distribution of the 40 ART surveyed ART sites in 2010. The following referred to the 10 different geographical regions in Cameroon: Adamaoua, Centre, Est, Extrême-Nord, Littoral, Nord, Nord-Ouest, Ouest Sud, and Sud-Ouest. The other names referred to the 40 ART sites enrolled in this survey. ART: Antiretroviral therapy.

All the sites selected for the survey in 2010 took part in the study, giving a participatory rate of 100%. The geographical distribution of the sites over the national territory suggests that the results generated represent the overall national paradigm, as shown in figure 1.

The national ART guidelines for the management of HIV infection were available in all the surveyed sites. The protocols for first line ARV drugs were also available and prescribed in all the sites. However, protocols for second line ARV drugs were prescribed only in the ATCs. In some of the study sites, ARV drugs were provided at the main pharmacy or at an additional pharmacy located within the health facility. Nevertheless, in all the study sites, management of ARV drugs was done solely with stocks forms, and most often by a pharmacy clerk. For a total of 55,646 patients on ART (among which 13,768 newly enrolled on ART) in the 40 sites, an overall heavy workload (7/200 staff-to-patient ratio) was found, and especially for the community relay agents who were often less operational even when physically present (see table 1).

**Table 1:** Staff/Patient ratio in the overall study population.

HealthStaff	Medical Doctor	Nurse	Diagnostician	Pharmacist or Pharmacy Clerk	Counselor	Community Relay Agent	Data Manager
Staff/Patient ratio	1/267	1/74	1/434	1/694	1/434	1/323	1/1387
Staff (min-max)	0–25	0–153	0–25	0–9	0–12	0–8	1

### The Level of Early Warning Indicators (EWI) in the Study Sites

Data abstraction and validation was effective for EWI_1, 2 and 3_ in all of the 40 sites. However, for EWI_4_, data from three sites were not validated due to incoherence (Nkongsamba Regional Hospital, “Saint-Jean de Malte” hospital and Douala General Hospital, all of the three sites belonging to the Littoral region of the country). For EWI_5_, we recorded one not validated (Pete Regional Hospital in the Far North region) and one unavailable data (Edéa District Hospital in the Littoral region). Thus, in terms of data collection and management, a unique region (Littoral region) was reported with major weakness. The detailed site performance from EWI_1_ to EWI_5_, grouped per region, is shown in table 2.

**Table 2:** Evaluation of EWIs in each of the 40 surveyed sites during the year 2010.

Region	Name of the ART site	EWI 1(Required target: 100%)	EWI 2(Required target: ≤20%)	EWI 3(Required target: ≥70%)	EWI 4(Required target: ≥90%)	EWI 5(Required target: 100%)	Regional Performance (% acceptable target)
Adamawa	*Hop Protestant Luth N’déré*	**100% (110/110)**	77% (84/110)	19% (21/110)	19% (21/110)	**100% (12/12)**	**30% (3/10)**
	*Hop Régional de N’déré*	**100% (145/145)**	46% (66/144)	49% (71/145)	23% (34/145)	42% (5/12)	
Centre	*Hop Central Ydé*	**100% (180/180)**	39% (64/165)	55,3% (94/170)	32% (58/180)	67% (8/12)	**36% (14/39)**
	*CHE/CNPS*	99% (158/160)	**20% (20/132)**	56,3% (90/160)	37% (58/157)	**100% (12/12)**	
	*HD Mbalmayo*	**100% (96/96)**	49% (46/93)	40,6% (39/93)	35% (30/86)	**100% (12/12)**	
	*HD Mfou*	**100% (98/98)**	47% (41/87)	49,5% (46/93)	20% (20/102)	58% (7/12)	
	*Hop. Gynéco Pédiatrique Ydé*	**100% (100/100)**	**4% (4/100)**	**85% (83/98)**	73% (73/100)	**100% (12/12)**	
	*HD Cité Verte*	**100% (100/100)**	51% (51/100)	41,6% (45/108)	NV	**100% (12/12)**	
	*Hop. Jamot Ydé*	**100% (155/155)**	43% (60/139)	50% (73/145)	56% (86/153)	75% (9/12)	
	*Hop Général de Ydé*	**100% (135/135)**	35% (47/135)	56,3% (76/135)	29% (39/135)	58% (7/12)	
Far North	*CTA HR Maroua*	**100% (155/155)**	39% (55/141)	44% (66/149)	12% (19/155)	92% (11/12)	**23% (3/13)**
	*HD Kousseri*	**100% (75/75)**	45% (28/66)	42,5% (31/73)	8% (6/75)	92% (11/12)	
	*HR Annexe de Pete*	**100% (100/100)**	56% (44/92)	35% (32/92)	NV	NV	
East	*HR Bertoua*	**100% (145/145)**	**10% (14/145)**	**90% (130/145)**	6% (8/144)	50% (6/12)	**67% (10/15)**
	*HD Abong mbang*	**100% (110/110)**	**20% (22/110)**	**76% (69/91)**	29% (32/110)	**100% (12/12)**	
	*CMA Belabo*	**100% (38/38)**	22% (8/37)	**73,7% (28/38)**	11% (4/38)	**100% (12/12)**	
Littoral	*HR Nkongsamgba*	**100% (140/140)**	77% (104/135)	55,3% (72/138)	NV	**100% (12/12)**	**44% (12/27)**
	*HD Edea*	**100% (46/46)**	**7% (3/43)**	**70% (30/43)**	14% (6/43)	NA	
	*HG Douala*	99% (158/160)	38% (60/160)	36,5% (159)	50% (74/147)	**100% (12/12)**	
	*HD Nylon Doual*	**100% (175/175)**	58% (101)175)	44% (70/159)	12% (20/171)	**100% (12/12)**	
	*Hôp. St Jean Malte*	**100% (110/110)**	33% (36/108)	37,1% (39/105)	NV	**100% (12/12)**	
	*Hopital Laquintine, Douala*	99% (158/160)	33% (53/160)	59,6% (93/156)	34% (54/160)	**100% (12/12)**	
North	*HR Garoua*	**100% (155/155)**	40% (53/133)	46,7% (72/154)	3% (5/152)	75% (9/12)	**20% (3/15)**
	*Centre Médical de la SN, Garoua*	**100% (75/75)**	36% (21/56)	52,2% (36/69)	26% (18/69)	42% (5/12)	
	*HD Guider*	**100% (134/134)**	33% (33/99)	40,6% (54/133)	11% (14/128)	67% (8/12)	
North-West	*HR Bamenda*	**100% (172/172)**	41% (68/167)	49% (82/167)	34% (50/146)	17% (2/12)	**20% (4/20)**
	*HD Ndop*	**100% (127/127)**	29% (36/126)	57,5% (73/127)	29% (36/126)	0% (0/12)	
	*Mbingo baptist Hops*	**100% (100/100)**	32% (30/95)	60% (62/104)	43% (45/104)	50% (6/12)	
	*Poly clinic Mezzam*	**100% (114/114)**	25% (28/114)	53% (62/117)	21% (26.125)	42% (5/12)	
West	*HR Bafoussam*	99% (153/155)	29% (44/150)	62% (96/155)	17% (27/156)	**100% (12/12)**	**35% (7/20)**
	*HD Foumbam*	**100% (140/140)**	22% (28/130)	**71% (95/134)**	1% (2/140)	**100% (12/12)**	
	*HD Bagangté*	**100% (56/56)**	39% (18/46)	42% (23/55)	23% (17/75)	50% (6/12)	
	*HD Dschang*	**100% (72/72)**	35% (24/69)	54% (39/72)	28% (28/99)	**100% (12/12)**	
South	*HR Ebolowa*	**100% (110/110)**	38% (41/110)	53,6% (59/110)	16% (17/108)	92% (11/12)	**40% (6/15)**
	*HD Ambam*	**100% (75/75)**	**17% (13/75)**	**82,7% (62/75)**	39% (29/75)	92% (11/12)	
	*HD Kribi*	**100% (100/100)**	42% (39/93)	49% (49/100)	39% (38/97)	**100% (12/12)**	
South-West	*CTA Limbe*	**100% (160/160)**	**19% (30/160)**	44,5% (68/153)	0% (0/160)	42% (5/12)	**40% (8/20)**
	*Baptist Hops Mutengene*	**100% (124/124)**	28% (34/128)	24,2% (30/124)	39% (44/114)	**100% (12/12)**	
	*HD Kumba*	**100% (135/135)**	32% (41/129)	46% (60/131)	7% (9/135)	17% (2/12)	
	*HR Buea*	**100% (110/110)**	**14% (13/90)**	**71,6% (73/102)**	25% (27/109)	25% (3/12)	

In *“* ***Bold*** *”*: EWI reaching the required target. NV: Not Validated. NA: Not Available.

EWI: Early Warning Indicator.

EWI_1_: Percentage of patients initiated on an appropriate first line ARV drug regimen, following national guidelines (Required target performance: 100%);

EWI_2_: Percentage of patient lost to follow-up after 12 months of enrolment to ART (Required target performance: ≤20%);

EWI_3_: Percentage of patient retained on appropriate first line ART after 12 month of treatment (Required target performance: ≥70%);

EWI_4_: Percentage of patients picking-up their ARV drugs on-time at the pharmacy of the ART site (Required target performance: ≥90%);

EWI_5_: Percentage of months without ARV drug shutdown at the pharmacy of the ART site (Required target performance: 100%).

For a general evaluation of the national ART roll-out program performance, data analysis of the overall national EWIs reported only the prescribing practices closed to the target, as shown in table 3.

**Table 3:** Overall national ART performance for each EWI.

Early Warning Indicators	Required target	Sites meeting the target (%)
EWI_1_: Good ARV prescribing practices (Percentage of patients initiated on an appropriate first line ARV drug regimens)	100%	90%(36/40)
EWI_2_: Lost to follow-up (% patient lost to follow-up after 12 months of enrolment to ART)	≤20%	20%(8/40)
EWI_3_: Retention on first line ART (% patient retained on first line ART after 12 month of ART)	≥70%	20%(8/40)
EWI_4_: On-time drug pick-up (Percentage of on-time ARV drug pick-up by the patient)	≥90%	0%(0/37)
EWI_5_: Drug supply continuity (Percentage of month without drug shutdown at the pharmacy)	100%	45%(17/38)

For EWI_1_, a national performance of 90% (36 out of 40), with the four failing sites having a performance of 99% (acceptable target is 100%), implies that ARV drugs prescribing practices in the country are in conformity with the current national ART guidelines. Of note, the four sites with below targeted performance were ATC (administering both first and second line ARV drugs), thus dealing with a heavy workload and complexity in their clinical routine activities. These sites belong to the centre, littoral and western regions, which are regions with the highest number of patients on treatment.

For EWI_2_, only 20% (8/40) of the sites were reported with a rate of lost-to-follow-up below acceptable target of ≤ 20%, representing 33% (4/12) and 14% (4/28) of the ATC and the HMU, respectively. This observation indicates a high rate of patient lost-to-follow-up nationwide; with ATCs performing about twice as well as HMUs. The poor performance ranged from 22% to 77%, with the Ngaoundéré Lutheran Presbyterian hospital (Adamawa region) and the Nkongsamba Regional Hospital (Littoral region) showing the highest number of lost patients (77%), while the Yaounde Gyneaco-Obstetric and Paediatric Hospital (Centre region) and the Edéa District Hospital (East region) recorded the lowest rates (4%, and 7% respectively). Furthermore, regional comparison showed that the East region had the best performance (Bertoua Regional Hospital, and Abong-Mbang District Hospital) while the Adamawa Region had the lowest (Ngaoundéré Lutheran Presbyterian Hospital). Therefore, there are several ART sites in need of in-depth investigations to accurately highlight the outcome of patient lost-to-follow-up (death, transfer, treatment interruption, etc). These parameters are crucial to evaluate the real impact of this indicator on patient compliance, as well as on the emergence and transmission of HIVDR strains at the local and national level.

For EWI_3_, only 20% (8/40) of sites succeeded in retaining patients on an appropriate first line ART following the acceptable performance of ≥70%, amongst which 17% (2/12) and 21% (6/28) were ATC and HMU, respectively. Therefore, there was a similar performance between ATC and HMU in terms of retaining patients on first line ART. However, sites with poor performances varied from 62% (Bafoussam Regional Hospital) to 19% (Ngaoundéré Lutheran Presbyterian Hospital). Furthermore, regional comparison revealed that the East region (all three sites) had the best performance while the poorest resulted from the Adamawa region (still the Ngaoundéré Lutheran Presbyterian Hospital, as previously observed in EWI_2_). This low rate of patient retention on first line may indicate an important switch from first to second line drugs, potentially due to a rapid spread of HIVDR within the geographical settings concerned.

For EWI_4_, none of the sites reached the required target of ≥90%, the rates obtained ranging from 0% at the Limbe Regional Hospital to 73% at the Yaoundé Gyneco-Obstetric and Paediatric Hospital. Comparison at the regional level showed that the Centre region performed best (41.5%), while the Far North region was the lowest (10%). Even though patient delay could be explained by the availability of drugs on the appointment day, these observations showed that the level of patient awareness and/or adherence to ART is very low in all the study sites, possibly requiring intensification or revision of adherence education, implementation of policies to reduce the waiting time at the pharmacy, a decrease in work load, or improved infrastructure.

For EWI_5_, only 45% of sites succeeded to ensure a 100% performance in supplying drugs without interruption in any of the ARV molecules. The 21 ART sites with poor performance represented 100% (11/11) of the ATC and 37% (10/27) of HMU. Thus, within the Cameroon context, ATC would be at three times higher risk to run out of stock as compared to HMU. A comparative regional performance showed that the Littoral was the only region to reach the required target of 100%, while the North-West region reported the lowest performance (27%), remarkably with the Ndop District Hospital showing drug stock-out every month. The national drug supply chain and/or the site drug management system need to be revised, especially in ATUs nationwide and in the North-West region of the country in particular.

## Discussion

An efficient evaluation of a national ART rollout programme in resource-limited settings like Cameroon requires the design and setting-up “easy-to-use”, effective and affordable strategies for the surveillance and prevention of HIVDR. More specifically, such strategies are of great asset in a context where the commonly available antiretrovirals used for patient management are of low genetic barriers. Thus, preventing HIVDR through EWI surveys may be useful in sustaining efforts to ensure the efficacy of the commonly used ARV drugs, especially with the rapid scale-up observed since 2003 after the “3 by 5” initiative and the ongoing universal access to treatment [6]–[7]. The present study was achieved due to the use of national standardized ART registers, the training of clinicians and data managers, and site supervision. However, the cases of discrepancies or incoherence of data management observed in a few sites suggest the need for continuous training of staff. Only an electronically standardized data management system could enable an early identification and correction of such incoherencies in data management, an activity that may negatively impact the monitoring and evaluation strategy toward a better control of HIVDR [5].

Our results show that good prescribing practices are performed in the all the ART sites within the country health system. This good performance would be likely due to the ongoing efforts in training clinicians prescribing ARV drugs launched several years ago. Importantly, patient initiation on ART is done by a consortium agreement during a *“treatment committee”* (a weekly meeting made-up of clinicians, nurses, pharmacists/pharmacy clerks, biologists/laboratory technicians, counselors, and/or community relay agents, all trained and involved in patient follow-up) at the ART site (ATC or HMU), an initiative which has greatly contributed to the conformity of prescribing habits with national guidelines. Therefore, an effective prevention of HIVDR goes along with staff training on HIV/AIDS therapeutic management. Our performance observed for EWI_1_ is consistent with those of Hedt et al in Malawi and of Hong et al in Namibia [18]–[19], even though the low sample size and incoherence in some of their data have likely restricted the public health relevance of their findings.

Despite compliance with EWI_1_, the risk for HIVDR emerging remains very high in the majority of the ART sites, as confirmed by the high rate of lost-to-follow-up, the low rate of patient retention on first line ART after 12 months, and the alarming delay observed in drug pick-up. Interestingly, ATCs performed about twice as well as HMUs (33% of ATCs met EWI_2_ compared to 14% of HMUs), suggesting that standard-meeting ATCs could mentor their respective HMUs, by sharing positive experiences that help in minimizing lost-to-follow-up. Evaluating the impact of the distance between the patient’s residence and the ART site, the patient educational level and compliance to ART, the impact of stigma on adherence, as well as the length of waiting time spent by a patient prior to medical consultation and/or reception at the pharmacy, may help out to improve patient’s adherence to the ART rollout program. It is obvious that, among patients lost to follow-up or on treatment interruption, there is a high risk of developing HIVDR; this is also an important factor in the transmission of resistant species in the community [17]. Also, even though ARV drugs are freely offered to patients in Cameroon, the costs for medical consultation and for laboratory analysis are potential limiting factors of patient access to care, thereby increasing the probability of a patient to be lost to follow-up. It is worth knowing that a patient lost to follow-up is considered as a potential HIVDR carrier [5]. Thus, additional resources and new community-based approaches may be needed to reduce the rate of missing patients in the national ART program. In their studies, Hong et al in Namibia had 89% performance, while Hedt et al found a performance of 84% and 14%, respectively from ART sites in the public and the private sector for EWI_2_ (required target: ≤20%) [18]–[19]. Interestingly, our data also showed an overall better performance for EWI_2_ in the public sector sites (23%, 8/35) as compared to the privates (0%, 0/5), thus suggesting that the public sector likely has more appropriate resources or approaches that increase target compliance. However, since a similar approach is used nationwide for patient management and follow-up, reducing the rate of patients lost to follow-up would require more resources allocated in the private sector. Further investigations may be needed for policy implementation.

The low rate of patient retention on first line ART after 12 months, reflecting a switch to a second line ART, may need to be confirmed by a detailed evaluation of patients really in need of a second line (through genotyping for HIV drug resistance testing). Such investigation would be of great asset to manage and to prevent early treatment failure, an ideal approach to limit the spread of resistance to the limited options of ARVs available within the national context, as it is the case in several resource-limited settings. The impact of delay in drug pick-up may be better analyzed by pill count, in order to effectively conclude on the real effect of the observed delays on ART observance, since patient’s appointments in several ATC and HMU are planned for 3 to 5 days before the entire consumption of drugs. The heavy workload, expressed by the staff-to-patients ratio (7/200), would have negatively affected the performance of EWI 2, 3 and 4 (these three are indicators related to ART adherence). With the creation of about a hundred new ART sites in the coming years [20], decentralization or task shifting in patient management (delineation of certain routine medical activities to trained nurses) and an active community engagement, would reduced the workload and the waiting time for patients, would increased patient adherence, and would finally be of great asset to control the development and spread of HIVDR [21].

The high rate of discontinuity in drug supply (observed in 55% of the sites) would have increased poor adherence to the ART programme, in terms of treatment interruption, lost to follow-up, and delay in drug pick-up. Even though alternative therapeutic options were generally provided during stock shutdown, a risk of emerging HIVDR still remains. Difficulties related to ARV continuous supply could be resolved by revising the drug supply system/mechanism and by involving more trained staff in drug procurement, or by decentralization in drug management.

Only a comprehensive intervention (involving an active participation and collaboration between the patients, the healthcare providers, and the community) will tackle the above mentioned weak points and challenges of the ART programme, and help in retaining patients as long as possible on first line ARV drugs available in the country [8]. Also, ensuring the availability of standardized ART registers in every ART site (ATC or HMU) is of great asset to the monitoring-evaluation process of the program and its strength in minimizing the burden of drug resistant patterns. During this study, we also observed a lack of regular supervision. Therefore, increasing the frequency of on-site supervision may also be of support in preventing HIVDR using EWIs. The national strategy toward ART adherence may need to be revised. Also, the role and positioning of community relay agents would be of paramount importance in the identification of lost to follow-up. In this prospect, improving communication strategies on ART adherence is necessary. Lastly, an active mentorship of HMU by an ATC is recommended, taking into consideration the key role of ARV “*treatment committee”*, as well as the required skills for routine data collection, analysis and on-site result exploitation of EWIs [5], [17]. Provision for staff continuous training could ensure rapid corrective measures, improve patient management and thus optimize on-site and the national HIVDR surveillance and prevention system.

In conclusion, the level of EWIs for HIVDR in Cameroon during the year 2010 indicates major challenges faced by the national ART programme in its effort to limit the spread of HIVDR. Despite the good ARV prescribing practices, indicators related to patient adherence and drug supply were very poor, thus reflecting the important risks of emerging HIVDR. Reducing the workload with task shifting, together with a better community engagement and regular site supervision, are key components to set-up efficient HIVDR preventive strategies.
